# Different Internal Fixation Methods for Sanders Type II and III Calcaneal Fractures: A 5‐Year Retrospective Study and Finite Element Analysis

**DOI:** 10.1111/os.14359

**Published:** 2025-01-18

**Authors:** Dewei Kong, Zhen Yang, Xinbin Fan, Ming Wu, Chao Song, Yan Zhang

**Affiliations:** ^1^ Postgraduate Training Base at Shanghai Gongli Hospital Ningxia Medical University Shanghai China; ^2^ School of Gongli Hospital Medical Technology University of Shanghai for Science and Technology Shanghai China; ^3^ Orthopedics Department Gongli Hospital of Shanghai Pudong New Area Shanghai China

**Keywords:** AOFAS, calcaneal fractures, finite element analysis, Sanders

## Abstract

**Objective:**

Soft tissue defects and postoperative wound healing complications related to calcaneus fractures may result in significant morbidity. The aim of this study was to investigate whether percutaneous minimally invasive screw internal fixation (PMISIF) can change this situation in the treatment of calcaneal fractures, and aimed to explore the mechanical effects of different internal fixation methods on Sanders type III calcaneal fractures through finite element analysis.

**Methods:**

This retrospective analysis focused on 83 patients with Sanders II and III calcaneal fractures from March 2017 to March 2022. Among them, 32 patients underwent PMISIF, 24 patients underwent tarsal sinus incision plate internal fixation (TSIPIF), and 27 patients underwent extended lateral incision plate internal fixation (ELIPIF). The present study aimed to compare various parameters, including the perioperative hospital stay, intraoperative blood loss, operative time, postoperative drainage volume, incidence of postoperative wound complications, and Gissane angle and Bohler angle data before surgery, after surgery, and at the last follow‐up, among the three treatment groups. Additionally, three different finite element models were created to simulate Sanders III calcaneal fractures treated with PMISIF, TSIPIF, and ELIPIF. The models were subjected to longitudinal stresses of 350 and 700 N, and the displacement and stress distribution were analyzed to compare the stability of each model.

**Results:**

Compared with ELIPIF and TSIPIF, PMISIF has several advantages, including shorter operative times, smaller incisions, shorter hospital stays, and lower incidences of postoperative complications. At the 12‐month time point after the operation, the percentages of patients with excellent and good American Orthopedic Foot and Ankle Society (AOFAS) functional scores were 96.9%, 91.7%, and 96.2%, respectively, for the three methods, demonstrating similar outcomes. Intraoperative blood loss in the PMISIF group was comparable to that in the TSIPIF group and lower than that in the ELIPIF group. There were no significant differences in the Gissane or Bohler angles among the three groups before or after the operation. However, the differences in the Gissane and Bohler angles after the operation within each group were statistically significant compared with those before the operation. Finite element analysis revealed that stress in all three internal fixation models was primarily concentrated on the subtalar articular surface, whereas displacement was mainly observed on the medial side of the subtalar articular surface. The peak stress and displacement of bone fragments and implants in the PMISIF model were lower than those in both the TSIPIF and ELIPIF models.

**Conclusion:**

PMISIF can achieve excellent and good rates comparable to those of TSIPIF and ELIPIF. Additionally, this approach offers the advantages of reduced operative trauma, a lower incidence of complications, and shorter preoperative preparation and hospitalization times. Furthermore, this approach can achieve a similar level of biomechanical stability.

## Graphical abstract

Our study demonstrated the effectiveness of minimally invasive percutaneous screw fixation for the treatment of Sanders II and III calcaneal fractures from both clinical and biomechanical points of view through retrospective and finite element analysis. It has better perioperative indicators and lower wound complications in the condition of stability comparable to the two types of plate internal fixation.

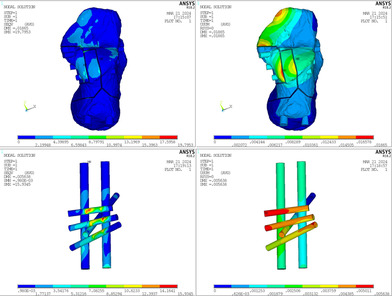

## 1 Introduction

Calcaneal fractures are a prevalent and common condition [1]. The traditional method of using a lateral “L” incision combined with plate internal fixation for treating calcaneal fractures has yielded satisfactory clinical outcomes. However, due to the thinness of the lateral calcaneal skin, inadequate blood supply, and high swelling tension of the fractured skin, the waiting period prior to surgery is prolonged. Additionally, postoperative complications such as poor wound healing, infection, and even skin flap necrosis frequently occur [2, 3]. Given these circumstances, methods to enhance perioperative indicators for treating calcaneal fractures and reduce the incidence of postoperative wound complications are urgently needed.

However, traditional plate internal fixation is widely considered the “gold standard” for the treatment of intra‐articular calcaneal fractures. However, some studies have suggested that the modified method may lead to a high postoperative wound complication rate of up to 31.2% [4], including complications such as wound infection, plate exposure, and sterile necrosis. Minimally invasive plate internal fixation is widely considered to compensate for the shortcomings of traditional plate internal fixation. However, some studies have suggested that the incidence of wound complications after minimally invasive plate internal fixation of bony sinus incisions is still as high as 17.6% [5]. In recent years, numerous scholars have employed a small incision through the tarsal sinus approach to directly visualize and reduce the articular surface area, in combination with minimally invasive plate internal fixation [6, 7]. This approach involves an incision of approximately 5 cm in length, extending from the tip of the lateral malleolus to the base of the fourth metatarsal. Traction, prying, pulling, and other techniques are utilized to achieve closed reduction of the calcaneal fracture. Additionally, alternative methods, such as external fixator fixation and balloon dilatation, are available. Percutaneous Kirschner wire fixation and screw fixation are also popular options. Compared with open reduction and internal fixation, this approach offers more advantages in reducing wound complications. To date, several studies, mainly case reports, have described various surgeries and conservative treatment options. However, there is a lack of uniform classification and treatment consensus, and only a few results have been supported by biomechanical evidence and reports.

Our previous study revealed that minimally invasive percutaneous cannulated screw internal fixation can achieve satisfactory results and reduce the incidence of wound complications, with no wound complications in 36 patients [8]. As a result, it remains unclear whether percutaneous cannulated screw minimally invasive fixation can achieve the clinical efficacy of plate internal fixation and whether the strength and stability of screw fixation and plate fixation are comparable. We hypothesized that compared with traditional plate fixation and minimally invasive plate fixation, cannulated screw fixation could provide satisfactory clinical efficacy and sufficient biomechanical stability.

To verify this hypothesis, the following research scheme was used: (1) A retrospective control method was used to study percutaneous hollow screw minimally invasive internal fixation, small incision plate internal fixation, and traditional “L” shaped incision plate internal fixation. The operation‐related indicators and imaging measurements were analyzed to prove the clinical feasibility of PMISIF. The clinical efficacy was evaluated. (2) Three finite element models of internal fixation for Sanders type III calcaneal fractures (conventional plates, minimally invasive plates, and minimally invasive screws) were used to evaluate and compare the biomechanical properties of the three internal fixation methods to demonstrate the mechanical stability of PMISIF. However, other methods, including external fixator fixation and balloon dilatation, are less commonly used in the clinic and have smaller ranges of application [9].

## 2 Materials and Methods

This study obtained ethical approval from the Medical Ethics Committee of Gongli Hospital of Shanghai Pudong New Area (2020–22). The project's design and execution strictly adhered to the principles of the CFDA/GCP and the Declaration of Helsinki. All methods employed in this study were conducted in accordance with relevant guidelines and regulations. Informed consent was obtained from all patients prior to surgery.

### 2.1 Patient Information

This retrospective analysis was performed on the clinical data of patients with Sanders type II and III calcaneal fractures from March 2017 to March 2022.

The inclusion criteria for patients were as follows: (1) Sanders type II and III calcaneal fractures, (2) percutaneous minimally invasive screw internal fixation (PMISIF), (3) tarsal sinus incision plate internal fixation (TSIPIF), (4) extended lateral incision plate internal fixation (ELIPIF), (5) follow‐up > 12 months, and (6) age > 14 years.

The exclusion criteria were as follows: (1) patients with open injury; (2) patients with other injuries; (3) patients without calcaneocuboid articular surface involvement; (4) patients requiring bone grafting; (5) patients with severe hypertension, hyperglycemia, coagulopathy, or other surgical contraindications; (6) patients with severe osteoporosis or severe obesity; and (7) patients with serious psychological problems.

### 2.2 Surgical Procedure

All patients underwent either continuous epidural anesthesia or general anesthesia. The operation was performed with the patient in the supine position. The steps of the procedure are outlined below.

#### 2.2.1 PMISIF Group

**FIGURE 1:**
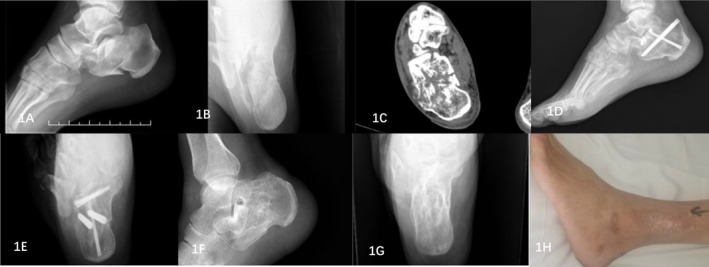
Imaging data of a typical PMISIF patient. (A) Preoperative calcaneal lateral x‐ray image showing that the articular surface of the calcaneus collapsed. (B) Preoperative axial x‐ray of the calcaneus revealed that the width of the calcaneus increased and that the length of the calcaneus shortened. (C) Preoperative MR image of the calcaneal fracture suggested a Sander type III fracture. (D) Postoperative calcaneal lateral x‐ray image showing calcaneal height recovery. (E) Postoperative calcaneal axial x‐ray image showing that the width and length of the calcaneus had recovered. (F) Lateral x‐ray films of calcaneus after removal of internal fixator showed calcaneus recovery. (G) Axial x‐ray films of calcaneus after removal of internal fixator showed calcaneus recovery. (H) The wound healed well after the operation.

The surgical procedure utilized a minimally invasive incision in the tarsal sinus. The incision was cut layer by layer 0.5–1.0 cm below the lateral malleolus of the fractured foot, with a length of 1.5–2.0 cm. The inner layer was bluntly separated to expose the subtalar articular surface. The possibility of injury to the sural nerve was reduced using a small incision, and a surgical draw hook was used to protect the sural nerve during articular surface reduction. The Gissane and Bohler angles were restored to normal by prying and repositioning the depressed articular surface. The calcaneal tuberosity was clamped with the bone tenaculum to pull the calcaneus backward and downward to correct the calcaneal varus deformity and restore the calcaneal length. The width of the calcaneus was restored by hammering or squeezing the lateral wall of the heel. After reduction, two cannulated screws were inserted inside and outside the insertion of the Achilles tendon, pointing toward the cuboid joint. A cannulated screw was placed at the lower part of the calcaneal tuberosity and was directed to the subtalar articular surface. One to three cannulated screws were placed from the lateral wall to the sustentaculum tali below the articular surface [8]. After x‐ray fluoroscopy confirmed the correct position, the wound was closed layer by layer, and a pressure dressing was applied. Figure 1 shows typical patients.

#### 2.2.2 TSIPIF Group

**FIGURE 2:**
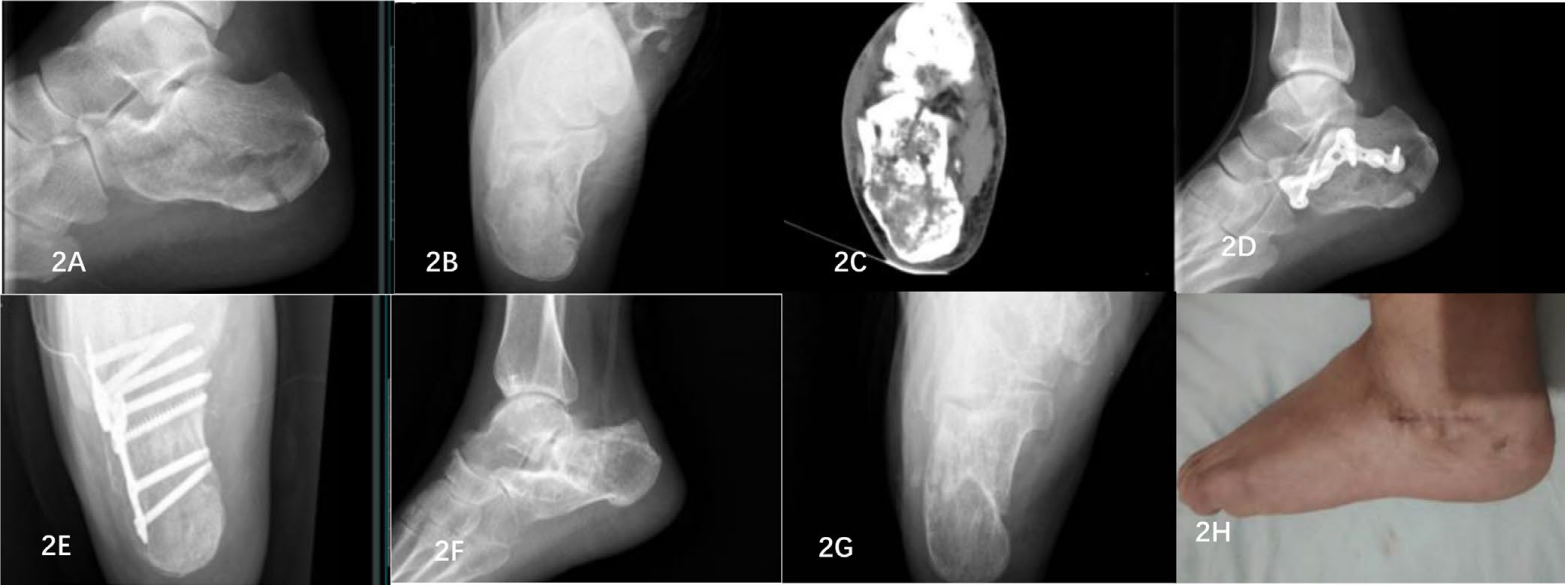
Imaging data of a typical TSIPIF patient. (A) Preoperative calcaneal lateral x‐ray image showing that the articular surface of the calcaneus collapsed. (B) Preoperative axial x‐ray of the calcaneus revealed that the width of the calcaneus increased and that the length of the calcaneus shortened. (C) Preoperative MR image of the calcaneal fracture suggested a Sander type III fracture. (D) Postoperative calcaneal lateral x‐ray image showing calcaneal height recovery. (E) Postoperative calcaneal axial x‐ray image showing that the width and length of the calcaneus had recovered. (F) Lateral x‐ray films of calcaneus after removal of internal fixator showed calcaneus recovery. (G) Axial x‐ray films of calcaneus after removal of internal fixator showed calcaneus recovery. (H) The wound healed well after the operation.

For surgery, a 3–4 cm long transverse incision was made in the tarsal sinus, and care was taken to protect the sural nerve and its accompanying blood vessels. Soft tissues were separated to expose the collapsed subtalar articular surface. The Gissane and Bohler angles were restored to normal by prying and repositioning the depressed articular surface. The calcaneal tubercle was clamped using a bone tenaculum, and the deformity was corrected through downward traction and eversion, restoring the calcaneal height and force line. The width of the calcaneus was subsequently restored by hammering or compressing the lateral wall of the heel. Once satisfactory reduction was achieved, the periosteal stripper was used to establish a subcutaneous channel behind the sheath of the peroneus longus and brevis tendon. The plate was placed below the posterior articular surface from the channel and fixed with screws. According to the principle of “three‐point fixation,” the fracture fragments of the calcaneus were fixed with plates and screws. The Steinmann pin and Kirschner wire were removed. C‐arm x‐ray machine fluoroscopy confirmed that the fracture had been reduced and that the internal fixation position was accurate. A negative pressure drainage tube was placed, which was sutured layer by layer, and a pressure bandage with an elastic bandage was used. Figure 2 shows typical patients.

#### 2.2.3 ELIPIF Group

**FIGURE 3:**
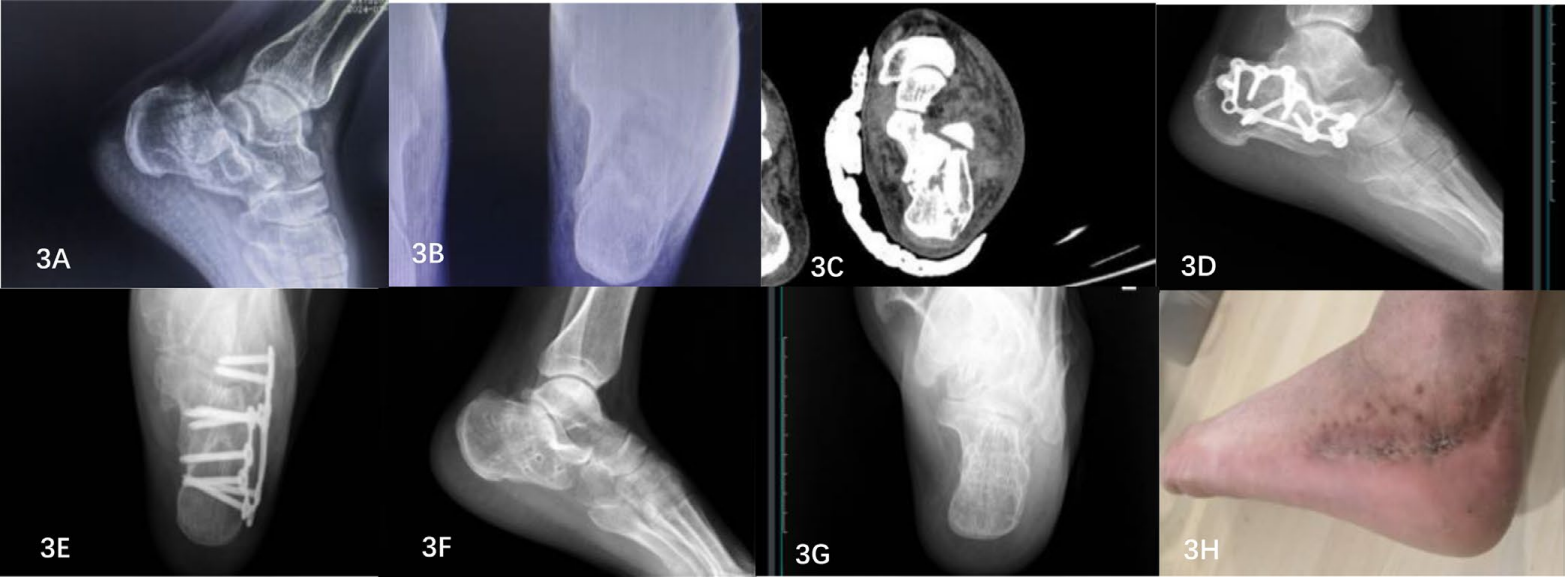
Imaging data of a typical ELIPIF patient. (A) Preoperative calcaneal lateral x‐ray image showing that the articular surface of the calcaneus collapsed. (B) Preoperative axial x‐ray of the calcaneus revealed that the width of the calcaneus increased and that the length of the calcaneus shortened. (C) Preoperative MR image of the calcaneal fracture suggested a Sander type III fracture. (D) Postoperative calcaneal lateral x‐ray image showing calcaneal height recovery. (E) Postoperative calcaneal axial x‐ray image showing that the width and length of the calcaneus had recovered. (F) Lateral x‐ray films of calcaneus after removal of internal fixator showed calcaneus recovery. (G) Axial x‐ray films of calcaneus after removal of internal fixator showed calcaneus recovery. (H) The wound healed well after the operation.

A lateral “L” incision was used for the surgical procedure. A long incision was made from 3 to 4 cm above the lateral ankle tip along the anterior edge of the Achilles tendon to 2–3 cm below the lateral ankle tip, and then in an arc shape 1 cm below the proximal base of the fifth metatarsal bone. Under the periosteum, the lateral wall of the calcaneus was exposed through precise dissection. The flap, periosteum, and peroneal tendon sheath were elevated to provide full exposure of the calcaneus and the subtalar articular surface. For larger fractures, a Kirschner needle may be used for temporary fixation. By continuous traction, the calcaneal tuberosity was clamped with a bone tenaculum to assist in restoring the length of the calcaneus. Simultaneously, the width of the calcaneus was restored by compression from both sides toward the center. An osteotome or periosteal stripper was inserted beneath the collapsed fracture block to pry out the invaginated bone block on the fracture surface, thus restoring the normal curvature of the articular surface. Temporary fixation was achieved using Kirschner wires. Once the C‐arm x‐ray machine confirmed proper positioning of the calcaneus, a locking plate was affixed to the lateral wall of the calcaneus and secured with locking screws. The Steinmann pin and Kirschner wires were removed. C‐arm x‐ray machine fluoroscopy confirmed that the fracture had been reduced, the internal fixation position was accurate, and a negative pressure drainage tube was inserted. Layer‐by‐layer suturing was performed, followed by the application of an elastic bandage for pressure dressing. Figure 3 shows typical patients.

### 2.3 Postoperative Treatment

The surgical and perioperative data were meticulously documented. The Bohler and Gissane angles of the calcaneus were precisely measured and recorded before surgery, immediately after surgery, and during the final follow‐up. Patients in both groups received detailed instructions for elevating the affected limb after surgery and were advised to undergo symptomatic treatments such as analgesia and edema reduction. Patients were subsequently guided to initiate ankle functional exercises starting from the second day after surgery. Standard x‐ray images taken at 4–6 weeks after surgery indicated that the fracture had gradually healed, and the patient then began to walk with partial weight bearing of the affected limb assisted by crutches.

### 2.4 Observation Indicators

Surgical‐related parameters included sex, age, body mass index (BMI), operative duration, extent of bleeding, postoperative drainage volume, occurrence of postoperative wound complications, and length of hospital stay. Perioperative observation indices, such as perioperative hospital stay, blood loss, and postoperative wound complications, helped us understand the operative effect, operative risk and patient prognosis, evaluate the quality of medical treatment, and optimize the treatment plan.

To evaluate the functional recovery of patients with calcaneal fractures, routine clinical follow‐up assessments were conducted at 4 and 8 weeks and at 6 and 12 months after surgery. The purpose was to determine whether there was any malunion of the articular surface and record any associated complications. The American Orthopedic Foot and Ankle Society (AOFAS) Ankle Hindfoot Score was used to evaluate the functional outcomes at the 12‐month postoperative mark.

### 2.5 Statistical Analysis

All the data were analyzed with SPSS 24.0 software. BMI, operative duration, extent of bleeding, postoperative drainage volume, and other measurement data are expressed as *X* ± *S*. The data of the three groups were analyzed by one‐way analysis of variance, and the Tukey–Kramer test was used for pairwise comparisons between groups. Age, sex, length of hospital stay, AOFAS score, and other count data were analyzed by chi‐square test or Fisher's exact probability method. Statistical significance was set at *p* < 0.05.

### 2.6 Establishing the Finite Element Model

A 26‐year‐old healthy adult male volunteer weighing 70 kg and 177 cm in height was selected. All the experimental plans and objectives were fully explained, and informed consent was obtained from the volunteer.

The right ankle and foot of the volunteer were scanned by CT. Specifically, the ankle joint was maintained in a normal standing posture, and the scanning direction was from the top of the talus to the bottom of the calcaneus. The scanning equipment used was a 64‐slice spiral CT (Siemens, Germany), and the scanning layer thickness was 0.625 mm. In addition, the bed speed was 1.3 mm/s, and the tube current and voltage were 500 mA and 120 kV, respectively. Finally, the CT data of the foot bones were saved in DICOM format.

#### 2.6.1 Establishing the Three‐Dimensional Model

The foot bone CT data were imported into ITK‐SNAP 3.8.0 software (University of Pennsylvania, USA). The pixel gray values were used to segment the talus, calcaneus, cuboid bone, cuneiform bone, subtalar articular cartilage, and calcaneocuboid articular cartilage images. A triangular surface mesh reconstruction was subsequently performed, and the resulting STL format file was exported. All STL files were imported into Altair Hyper Works (Version 2020, Altair, USA).

**FIGURE 4:**
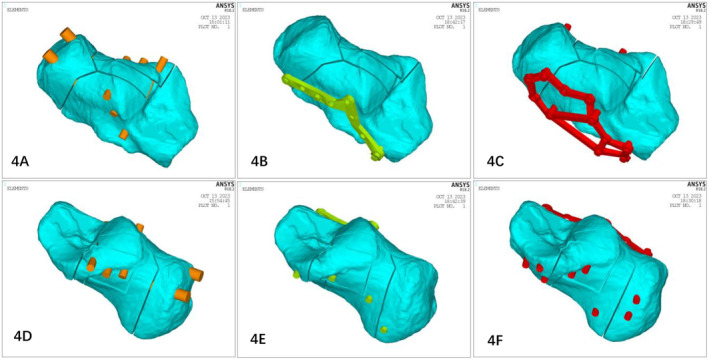
Schematics of the three internal fixation models. (A and D) Percutaneous minimally invasive screw internal fixation (PMISIF); (B and E) tarsal sinus incision plate internal fixation (TSIPIF); (C and F) extended lateral incision plate internal fixation (ELIPIF).

CT data were used to establish a calcaneal model. Following the definition of Sanders type III calcaneal fracture, the corresponding fracture model was generated. Three‐dimensional solid models of the traditional plate and minimally invasive plate and screws were established in HyperWorks based on size and clinical data of the implant. These models were assembled according to the surgical fixation method to generate different internal fixation models (Figure 4).

#### 2.6.2 Material Property Definitions

All parts of the model were assumed to be continuous, homogeneous, and isotropic linear elastic materials. The elastic modulus and Poisson's ratio of the cortical bone, cancellous bone, cartilage, and implants (three‐dimensional frame screws, traditional plates, and minimally invasive plates) were set [10, 11] (Table 1). Face‐to‐face contact was established between the bone block and the implant, and nonseparation contact was set between the fracture blocks.

**TABLE 1:** Material parameters of the three‐dimensional finite element model.

Material	Elastic modulus (MPa)	Poisson's ratio
Cortical bone	7300	0.3
Cancellous bone	100	0.3
Plate and screw	200,000	0.28
Cartilage	10	0.46

#### 2.6.3 Mesh Generation

The finite element mesh was divided using ANSYS 21.0 software. The meshing of the models was generated automatically by defining the node spacing on the sides of the bone block and implant. The node spacing and the optimal dimensions of the elements were selected based on the convergence test of the elements. The average mesh size of the calcaneus model was 1.3 mm, and the average mesh size of the implant model was 0.75 mm. In the fracture mesh model, there were 220,087 nodes and 1,130,782 finite elements. In the mesh model of the minimally invasive screw group, there were 233,590 nodes and 1,211,791 elements. In the mesh model of the minimally invasive steel plate group, there were 256,226 nodes and 1,338,565 elements. In the mesh model of the traditional steel plate group, there were 260,804 nodes and 1,360,086 elements.

#### 2.6.4 Setting the Model Conditions

The contact conditions for each component of the model were defined. Specifically, the screw and steel plate models were set as binding, the screw and calcaneus models were set as binding, and the cortical bone and cancellous bone models were set as binding. The connection between different assemblies was node coupling. The calcaneus, cuboid, and cuneiform bases were completely restricted in three‐way translation and three‐way rotation, ensuring that their displacements along the *X*, *Y*, and *Z* axes were completely limited. The load was applied at the top of the talus, exerting vertical pressure toward the distal end. Furthermore, the loading forces were set at 700 and 350 N to simulate the stress state of the calcaneus during walking or standing on one foot or both feet, respectively, with a weight of 70 kg.

#### 2.6.5 Validation of Model Validity

According to Mao Binyao et al. [12], the heel bears 46.68% of the plantar force. After loading 700 N of stress on the top of the talus, the displacement of the fracture model was 0.67 mm, and the force on the calcaneus was approximately 350 N. After loading 700 N of stress on the top of the talus, the displacement of the fracture model was 0.67 mm, and the force on the calcaneus was approximately 350 N, which was similar to the displacement of the fracture model of the calcaneus when B. Yu et al. [13] loaded 350 N of stress on the subtalar articular surface of the calcaneus (approximately 0.64 mm). Considering the differences in the models, the model in this study can be considered valid.

## 3 Results

The sample characteristics of the patients are detailed in Table 2. There were no significant differences in the demographic data among the three groups. A retrospective selection of 83 patients was made for this study. Patients were categorized into three groups according to the surgical method used: the PMISIF group (32 patients), the TSIPIF group (24 patients), and the ELIPIF group (27 patients). All patients were followed up for at least 1 year and up to 4 years.

**TABLE 2:** Basic patient data.

	PMISIF	TSIPIF	ELIPIF	*p*‐value
Patients (*n*)	32	24	27	
Classification				0.297
II	12	10	6	
III	20	14	21	
Sex				0.604
Male	28	20	25	
Female	4	4	2	
Age (mean ± SD, years)	54.27 ± 13.80	50.86 ± 13.00	45.53 ± 12.22	0.128
BMI (mean ± SD, kg/m^2^)	23.89 ± 3.48	24.26 ± 3.79	23.01 ± 2.74	0.555

### 3.1 Evaluation of the Clinical Curative Effect

All patients underwent successful surgery. The hospitalization and operative times in the PMISIF group were significantly better than those in the TSIPIF and ELIPIF groups, the intraoperative blood loss in the PMISIF group was less than that in the ELIPIF group, and there was no need to place a drainage tube after the operation (Tables 3 and 4). At 12 months after surgery, the percentages of excellent and good AOFAS scores were 96.9% in the PMISIF group, 91.7% in the TSIPIF group, and 96.2% in the ELIPIF group (Table 5). After the operation, there was one case of excessive wound exudation in the PMISIF group, one case of tension blisters and one case of excessive wound exudation in the TSIPIF group, and two cases of redness and swelling and two cases of excessive wound exudation in the ELIPIF group (Table 3).

**TABLE 3:** Perioperative data of the three groups of patients.

	PMISIF	TSIPIF	ELIPIF
Hospital stay (days)	9.24 ± 4.49	15.14 ± 7.35	18.63 ± 8.245
Operation time (hours)	123.73 ± 50.29	143.21 ± 68.45	149.88 ± 20.64
Preoperative bleeding (mL)	35.91 ± 26.71	59.64 ± 48.61	63.25 ± 52.77
Postoperative drainage volume (mL)	—	70.50 ± 50.64	88.53 ± 49.96
Incidence of postoperative complications	3.1% (1 case)	8.3% (2 cases)	14.8% (4 cases)

**TABLE 4:** *p* values for different surgical indicators between different groups.

Group	Hospital stay (days)	Operative time (hours)	Preoperative bleeding (mL)	Postoperative drainage volume (mL)
PMISIF vs. TSIPIF	0.06 (2.954)	0.042 (2.112)	0.067 (1.893)	—
PMISIF vs. ELIPIF	0.00 (4.435)	0.00 (4.777)	0.042 (2.110)	—
TSIPIF vs. ELIPIF	0.235 (1.213)	0.200 (1.312)	0.846 (0.195)	0.328 (0.994)

**TABLE 5:** AOFAS scores at 12 months after surgery.

	PMISIF	TSIPIF	ELIPIF	*p* value
AOFAS	89.41 ± 7.11	89.43 ± 9.21	90.24 ± 6.92	0.936
Excellent	15	14	15	0.701
Good	16	8	11	
Average	1	2	1	
Poor	0	0	0	

### 3.2 Imaging Results

**FIGURE 5:**
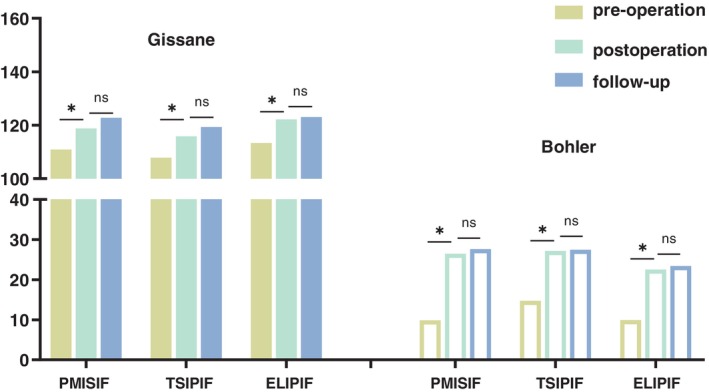
Comparison of the Gissane and Bohler angle data of the three groups before the operation, after the operation, and at the last follow‐up. PMISIF, percutaneous minimally invasive screw internal fixation; TSIPIF, tarsal sinus incision plate internal fixation; ELIPIF, extended lateral approach plate internal fixation (*, *p* < 0.05; ns, *p* > 0.05).

**FIGURE 6:**
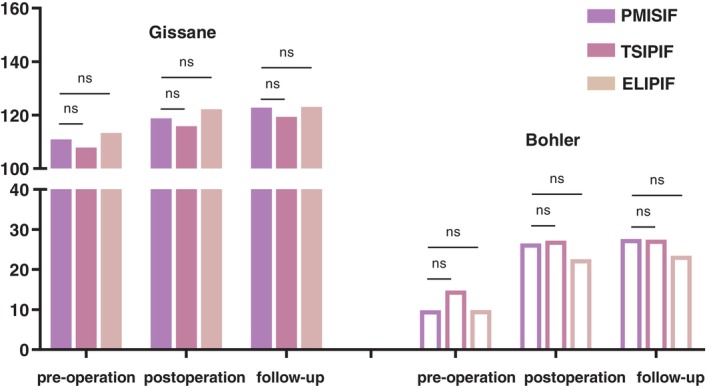
Comparison of the Gissane and Bohler angle data of the three groups at different time points. PMISIF, percutaneous minimally invasive screw internal fixation; TSIPIF, tarsal sinus incision plate internal fixation; ELIPIF, extended lateral approach plate internal fixation..

There were significant differences in the Gissane and Bohler angles among the three groups before and after the operation, but there were no significant differences after the operation or at the last follow‐up (Figure 5). In addition, there were no significant differences in the Gissane or Bohler angles among the three groups before surgery, immediately after surgery, or at the last follow‐up (Figure 6).

### 3.3 Results of the Finite Element Analysis of the Three Surgical Methods

#### 3.3.1 Model Validation

Ansys software was used to apply vertical stresses of 350 and 700 N to the three internal fixation models. The resulting displacement distances of the fracture block and internal fixation in different directions were compared. Upon measurement, the ranges of motion of the three finite element models were similar to that of the in vitro model. Considering the individual differences in the models themselves, the differences in the results were deemed acceptable. Therefore, the established finite element model could accurately simulate the biological structure of the calcaneus.

#### 3.3.2 Peak Displacements of the Finite Element Models (Figure 7)

**FIGURE 7:**
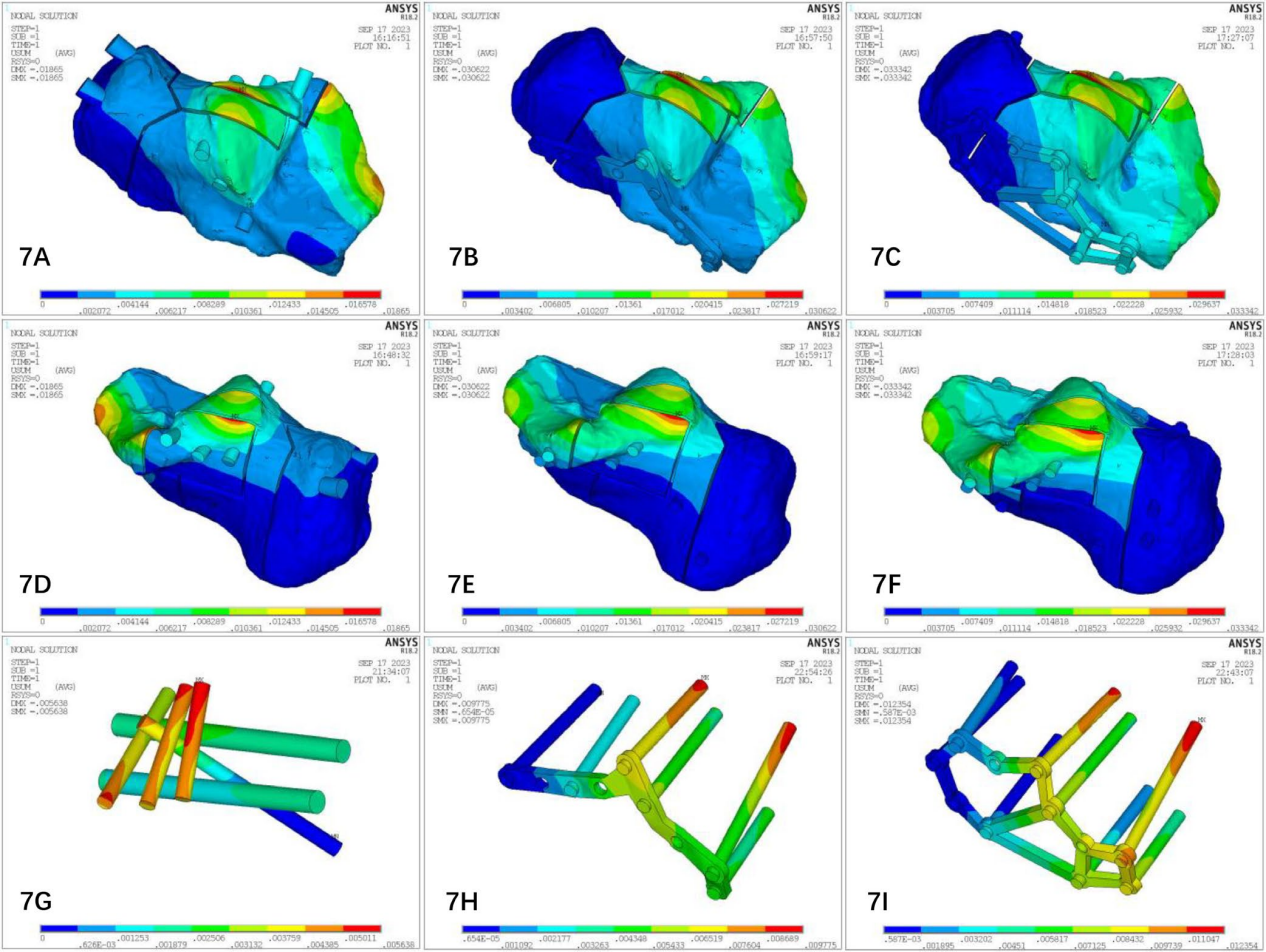
Displacement distributions of the three models. In the displacement distribution nephogram of the three internal fixation models, the red part indicates that the displacement distance is large, and the blue part indicates that the displacement is small or that there is no displacement.

##### 3.3.2.1 Model Displacements

The peak displacements of the models were as follows: the maximum displacement was observed on the subtalar articular surface of the calcaneus. At 700 N stress loading, the peak displacements of the fracture, PMISIF, TSIPIF, and ELIPIF models were 0.671 mm, 0.619 mm, 0.629 mm, and 0.632 mm, respectively. At 350 N of stress loading, the peak displacements of the fracture, PMISIF, TSIPIF, and ELIPIF models were 0.336 mm, 0.309 mm, 0.316 mm, and 0.314 mm, respectively.

##### 3.3.2.2 Displacement of the Internal Fixator

After the fracture block was removed using ANSYS, the peak displacements of the internal fixation were as follows: after loading at 700 N stress, the peak displacements of the PMISIF, TSIPIF, and ELIPIF models were 0.005638 mm, 0.009775 mm, and 0.012354 mm, respectively. After loading at 350 N, the peak displacements of the PMISIF, TSIPIF, and ELIPIF models were 0.002819 mm, 0.004887 mm, and 0.006117 mm, respectively.

#### 3.3.3 Von Mises Stress (VMS) of the Finite Element Models (Figure 8)

**FIGURE 8:**
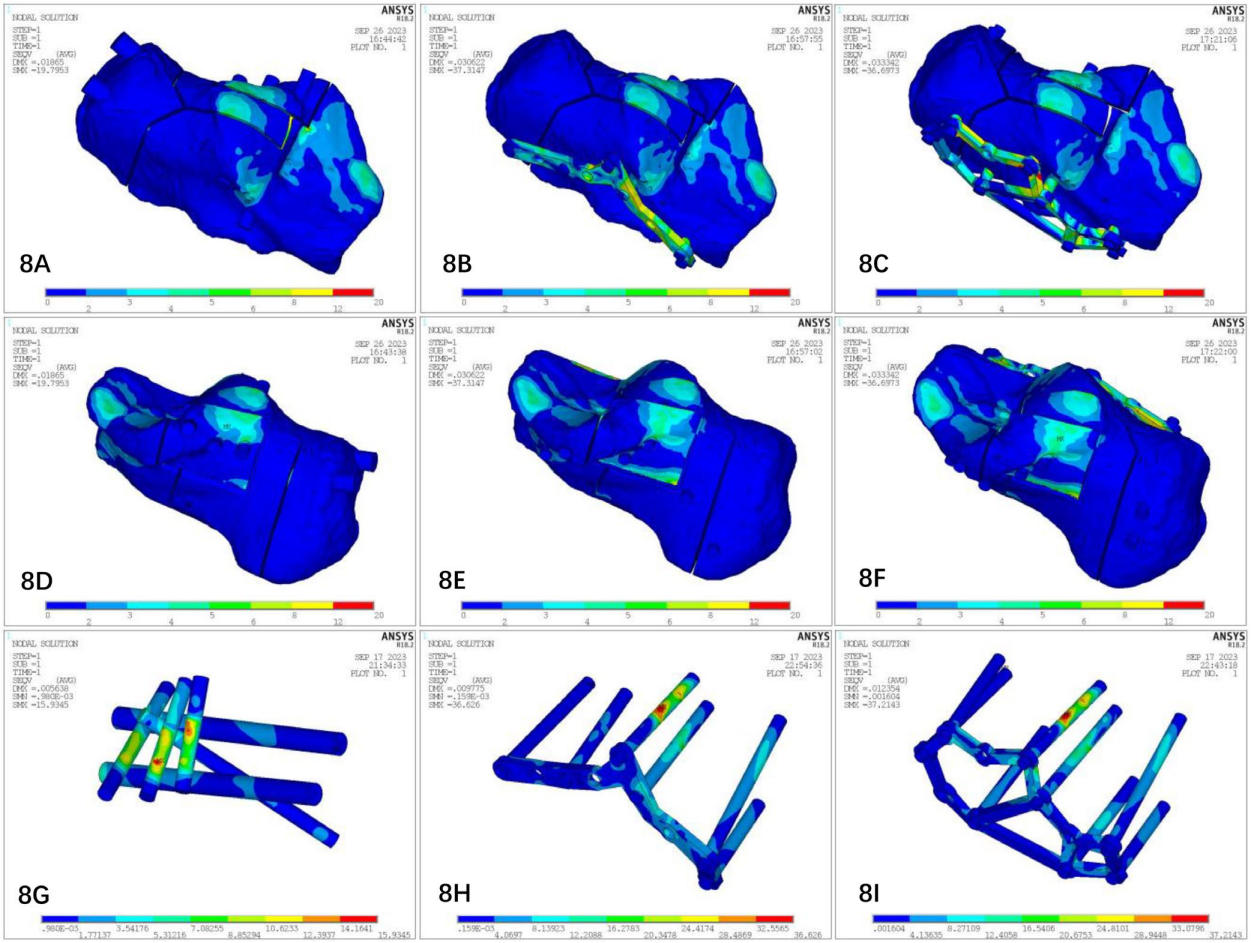
Stress distributions for the three models. In the stress distribution nephogram of the three internal fixation models, the red part indicates that the stress is greater and more concentrated, and the blue part indicates that the stress is lower and more scattered.

##### 3.3.3.1 Model Stress Distribution

The peak stresses of the model were as follows: the stresses were distributed mainly at the subtalar articular surface of the calcaneus. At 700 N stress loading, the peak stresses of the fracture, PMISIF, TSIPIF, and ELIPIF models were 25.363 MPa, 19.795 MPa, 37.315 MPa, and 37.438 MPa, respectively. At 350 N stress loading, the peak stresses of the fracture, PMISIF, TSIPIF, and ELIPIF models were 13.379 MPa, 9.898 MPa, 19.388 MPa, and 18.719 MPa, respectively.

##### 3.3.3.2 Stress Distribution of the Internal Fixator

After the fracture block was removed using ANSYS, the peak stresses of the internal fixator were as follows: at 700 N stress loading, the peak stresses of the percutaneous screw model, the minimally invasive plate model, and the traditional plate model were 15.935 MPa, 39.404 MPa, and 37.214 MPa, respectively. At 350 N stress loading, the peak stresses of the percutaneous screw model, the minimally invasive plate model, and the traditional plate model were 7.967 MPa, 19.702 MPa, and 20.021 MPa, respectively.

## 4 Discussion

Our study revealed that PMISIF is an effective method for the treatment of Sanders type II and III calcaneal fractures; moreover, it is comparable to the two types of plate internal fixation in terms of efficacy and superior to the two types of plate internal fixation in terms of perioperative‐related indicators. By analyzing the finite element model, we determined the deformation and stress change trends of Sanders type III calcaneal fractures under different internal fixation conditions. Although each of the three models has its own advantages and disadvantages, PMISIF can achieve the same fixation effect as the two plate fixation methods in terms of biomechanics.

### 4.1 The Current Reality of Lateral “L” Incisions and Tarsal Sinus Incisions

The traditional “L” type of extended lateral incision open reduction and plate internal fixation technique proposed by Bnirschke and Sangeorzan [14] in 1993 is considered the gold standard for the surgical treatment of calcaneal fractures. This approach provides sufficient exposure and space for reduction and plate placement. However, it also has several disadvantages. The long incision and thin flap of traditional surgery damage the soft tissue and microcirculation around the calcaneus, leading to complications such as poor wound healing, flap necrosis, wound infection, and osteomyelitis [15, 16, 17]. In addition, the swelling of the surrounding calcaneal tissue and poor soft tissue conditions after fracture render early surgery unsuitable, as it increases the risk of postoperative wound complications. Surgeons often choose to wait until the swelling subsides completely before surgery, but this waiting period increases the risk of lower extremity venous thrombosis and adds to the economic burden on patients. To address these issues, some scholars have proposed a modified minimally invasive plate technique through a tarsal sinus incision, which reduces the intraoperative wound range and postoperative complications [18]. However, this approach still requires the establishment of a subcutaneous pathway for steel plate implantation. Furthermore, to pursue postoperative primary healing, this approach does not fully expose the calcaneus, resulting in excessive traction of the incision and potential damage to the microcirculation of the surrounding soft tissue, including the risk of sural nerve injury [19].

### 4.2 PMISIF Has Obvious Advantages in Terms of Perioperative and Postoperative Complications

PMISIF involves a pin‐sized tarsal sinus incision that is only half the length of the TSIPIF. This procedure only necessitates prying reduction of the subtalar articular surface, eliminating the need for aggressive stripping of subcutaneous tissue to establish a surgical channel. This approach maximizes the preservation of soft tissue and the vascular network surrounding the calcaneus, while minimizing potential sural nerve damage during surgery. Compared with ELIPIF and TSIPIF, these attributes contribute to the advantages of PMISIF, such as reduced intraoperative trauma, expedited postoperative recovery, and a lower incidence of complications. Because of the minor surgical incision and high tolerance of skin conditions, patients do not need to suffer a long time before surgery to wait for the skin swelling to subside. A comprehensive retrospective clinical analysis revealed that the PMISIF could match the clinical efficacy of the gold standard “Plate Internal Fixation” based on the AOFAS score 12 months postsurgery. Moreover, PMISIF was superior to TSIPIF and TSIPIF in terms of mitigating perioperative and postoperative wound complications.

### 4.3 FEM Validation Revealed that the PMISIF Could Provide Satisfactory Biomechanical Stability

The main goal of surgical treatment for calcaneal fractures is to ensure the smoothness of the subtalar articular surface, restore the physiological Gissane and Bohler angles, and restore the relationship between the calcaneus and the talus [20, 21]. The loss of the normal relationship between the calcaneus and talus and the unevenness and displacement of the articular surface inevitably cause changes in the stress and movement of the subtalar joint. This severely affects the valgus function of the subtalar joint, resulting in secondary injuries such as traumatic arthritis of the subtalar joint and its surrounding joints, affecting the normal walking function of the foot [22, 23]. By observing the displacement results of the finite element analysis, we found that when the calcaneus was subjected to axial loading, the displacement and stress distribution of each fracture block gradually increased from the lateral to the medial side in a step‐like manner. The displacement of the three internal fixation models was distributed mainly at the subtalar joint surface, which was consistent with the study of Gültekin et al. [24]. Under 350 and 700 N stress compression, the maximum displacement of the PMISIF model was smaller than those of the two types of plate internal fixation, which has a better anti‐displacement effect. In the PMISIF model, a row of screw structures formed by the three transverse screws and the three screws placed from the calcaneal tuberosity crossed up and down to the sustentaculum tali stabilized all the fracture fragments. In this manner, the three longitudinal screws and the transverse row of the screw system of the subtalar articular surface formed a tetrahedral frame of the entire calcaneus in a three‐dimensional structure. The weakest neutral trigone of the calcaneus is located in the center of the tetrahedron [25], thus receiving the most powerful protection. From the perspective of biomechanics, PMISIF can achieve the anti‐displacement effect of plate internal fixation, which ensures that the subtalar articular surface will not collapse after reduction due to weight bearing or rehabilitation training. The traditional incision method has a slight advantage in restoring the flatness of the articular surface, which can achieve complete anatomical reduction of the articular surface under direct vision [26], whereas PMISIF can disperse the stress at the fracture site and better maintain the restored articular surface. In addition, it can be seen from the stress nephogram that the stress peak points of the three internal fixation models are located on the internal fixation device, indicating that the three internal fixation devices effectively share the stress of the fracture block, but the stress of the two plate internal fixation models is mainly concentrated on the top screw. The stress of the PMISIF device is distributed mainly on the three transverse screws on the subtalar articular surface, and the distribution is more even, which reduces the risk of failure of the internal fixation device. Based on clinical verification, this research group conducted a comparative analysis of PMISIF and tarsal sinus incision plate internal and traditional ELIPIF via finite element analysis of the mechanical stability of fixation. The deformation and stress trends of Sanders type III calcaneal fractures under different internal fixation conditions were determined. Each of the three methods has advantages and disadvantages; however, with respect to biomechanics, PMISIF, TSIPIF, and ELIPIF can achieve similar fixed effects.

### 4.4 Summary of the Advantages, Applications, and Limitations of PMISIF

In this study, we demonstrated that PMISIF can achieve the same clinical effects and mechanical stability as plate internal fixation, which is sufficient to meet the needs of both doctors and patients. Moreover, the operative method of PMISIF does not require obvious incisions to separate large areas of subcutaneous tissue, which provides soft tissue protection and results in less bleeding, and less stimulation of internal fixation devices. Moreover, it can reduce the incidence of postoperative complications and shorten the postoperative recovery time and thus is a more recommended surgical method. PMISIF can be clinically applied to patients with common Sanders type II and type III intra‐articular calcaneal fractures. However, the research team believes that PMISIF also has certain limitations. First, PMISIF may not achieve ideal reduction and fixation effects for overly complex and severe calcaneal fractures, especially those with a high degree of fracture mass comminution and severe articular surface collapse. Second, the doctor needs to have a high level of operational skills and extensive experience to perform the screw placement and fracture reduction accurately. Otherwise, problems such as improper screw placement and poor fracture reduction may occur.

### 4.5 Strengths and Limitations of This Study

The strengths of this research are that the finite element model was used to analyze the biomechanics of the calcaneus, and the mechanical characteristics of the three internal fixation models were comprehensively demonstrated. Second, the support mode of pure screw internal fixation on fractured calcaneus was simulated. The clinical efficacies of the two types of plate internal fixation and PMISIF methods were compared based on abundant data.

The limitations of this research lie in the simplification of the fracture line during the construction of the three‐dimensional finite element fracture model, which merely provides an approximate representation of the actual fracture. In fact, the fracture line of a calcaneal fracture is irregular, but it is sufficient to fulfill the research prerequisites. This experiment only simulated and analyzed standing states during static standing and one‐leg standing, and did not analyze the variable load change. However, the actual stress on the calcaneus is often the superposition of multiple loads. The effects of soft tissues such as ligaments and muscles around the ankle and the calcaneus on fracture stability were also not considered in this study.

## 5 Conclusion

Currently, there are controversies regarding the surgical choice for Sanders II and III calcaneal fractures in clinical practice. Through this study, the authors believe that PMISIF can achieve excellent and good rates comparable to those of TSIPIF and ELIPIF. Additionally, this approach offers the advantages of reduced operative trauma, a lower incidence of complications, and shorter preoperative preparation and hospitalization times. Furthermore, this approach can achieve a similar level of biomechanical stability.

## Author Contributions

Dewei Kong completed the writing of this manuscript. Dewei Kong, Xinbin Fan, Chao Song, and Ming Wu collected the calcaneal data, produced the model, and analyzed the model results. Dewei Kong, Xinbin Fan, and Zhen Yang completed the patient data collection and data analysis. Zhen Yang and Xinbin Fan proofread the manuscript. Yan Zhang designed the entire experiment and guided the process.

## Ethics Statement

This study was approved by the Ethics Committee of Naval Military Medical University Affiliated to Gongli Hospital.

## Conflicts of Interest

The authors declare no conflicts of interest.
